# The mysterious anelloviruses: investigating its role in human diseases

**DOI:** 10.1186/s12866-024-03187-7

**Published:** 2024-01-29

**Authors:** Mohammad Sabbaghian, Hamidreza Gheitasi, Ali Akbar Shekarchi, Ahmad Tavakoli, Vahdat Poortahmasebi

**Affiliations:** 1 Department of Bacteriology and Virology, Faculty of Medicine, Tabriz University of Medical Sciences, Tabriz, Iran; 2 Research Center for Clinical Virology, Tehran University of Medical Sciences, Tehran, Iran; 3 Department of Pathology, Faculty of Medicine, Tabriz University of Medical Sciences, Tabriz, Iran; 4 Research Center of Pediatric Infectious Diseases, Institute of Immunology and Infectious Diseases, Iran University of Medical Sciences, Tehran, Iran

**Keywords:** Anellovirus, Torque teno virus, Viral infection, Coinfection, microRNA

## Abstract

Anelloviruses (AVs) that infect the human population are members of the Anelloviridae family. They are widely distributed in human populations worldwide. Torque teno virus (TTV) was the first virus of this family to be identified and is estimated to be found in the serum of 80–90% of the human population. Sometime after the identification of TTV, Torque teno mini virus (TTMV) and Torque teno midi virus (TTMDV) were also identified and classified in this family. Since identifying these viruses, have been detected in various types of biological fluids of the human body, including blood and urine, as well as vital organs such as the liver and kidney. They can be transmitted from person to person through blood transfusions, fecal-oral contact, and possibly sexual intercourse. Recent studies on these newly introduced viruses show that although they are not directly related to human disease, they may be indirectly involved in initiating or exacerbating some human population-related diseases and viral infections. Among these diseases, we can mention various types of cancers, immune system diseases, viral infections, hepatitis, and AIDS. Also, they likely use the microRNAs (miRNAs) they encode to fulfill this cooperative role. Also, in recent years, the role of proliferation and their viral load, especially TTV, has been highlighted to indicate the immune system status of immunocompromised people or people who undergo organ transplants. Here, we review the possible role of these viruses in diseases that target humans and highlight them as important viruses that require further study. This review can provide new insights to researchers.

## Introduction

**Fig. 1:**
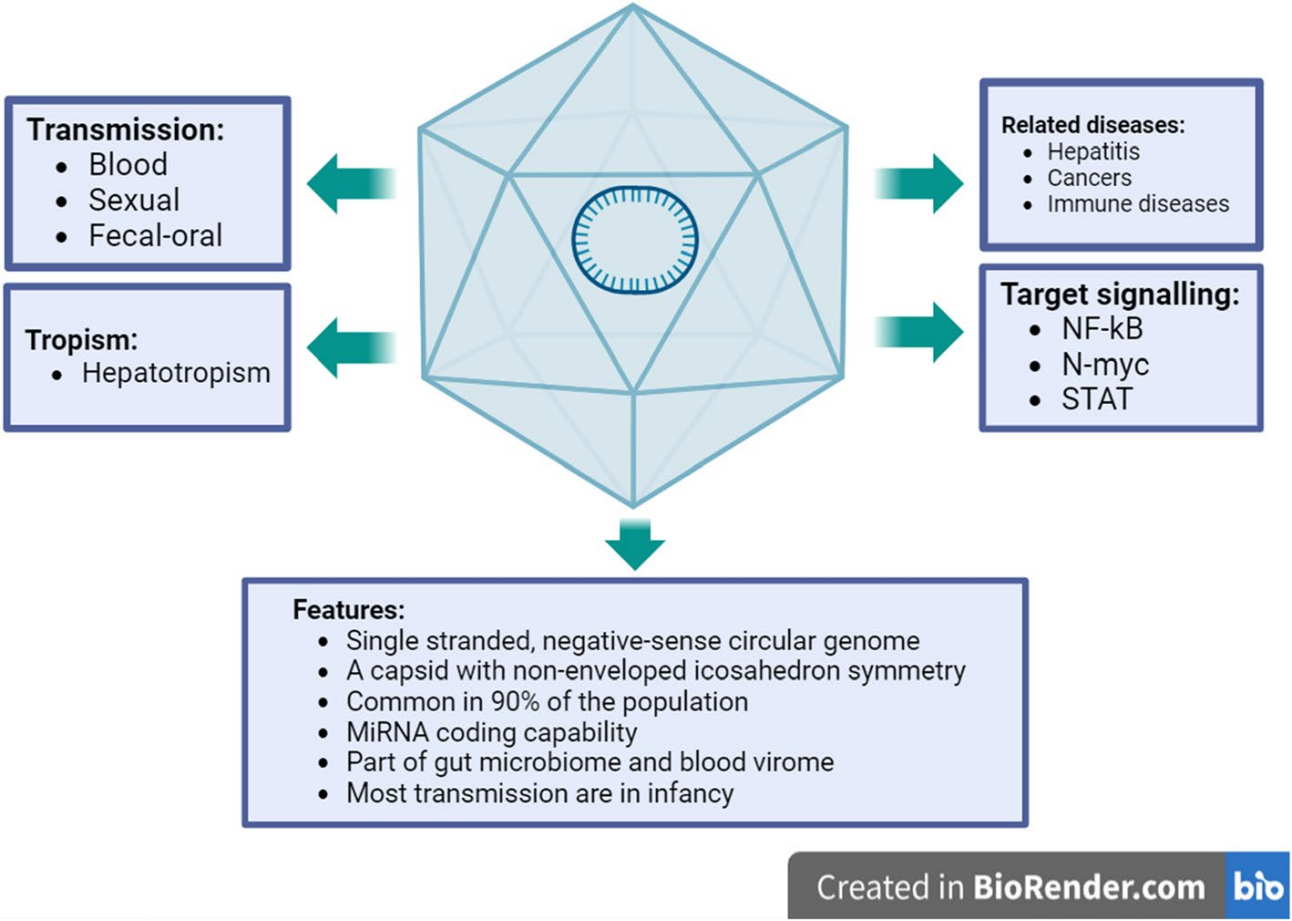
A figure containing a brief overview of the characteristics of anelloviruses infecting humans. Adapted from “Overview of the characteristics of anelloviruses”, by BioRender.com (2023). Retrieved from https://app.biorender.com/biorender-templates

The Anelloviridae family is a large viral family that includes many viruses that infect a wide range of animal species, including humans. This virus family consists of 30 different genera and 156 species. Until now, three genera of this viral family have been identified in humans, each with significant genetic diversity. Three genera of this family can infect humans and other vertebrates: alphatorquevirus, betatorquevirus, and gammatorquevirus, which include 26, 38, and 15 species, respectively. Torque teno virus (TTV) belongs to alphatorquevirus, torque teno mini virus (TTMV) belongs to betatorquevirus and torque teno midi virus (TTMDV) belongs to gammatorquevirus. The genome of these viruses is circular single-stranded, negative-sense DNA ranging from ∼2.0 to 3.9 kilobases (kb); the capsid of these viruses has icosahedron symmetry, and their total particle diameter is about 18 to 30 nm, and their nucleocapsid does not have a lipid envelope [1–6]. Human anelloviruses (AVs) are among the most common human viruses and are considered part of the human virome. They are found in approximately 90% of adult humans and infect the blood and most organs [3, 7]. They have been isolated from various biological samples such as saliva, urine, and bile, and this shows that they have a broad tissue tropism [8] (Table 1). AVs are transmitted and infect humans via a variety of routes, including sexual contact, blood transfusion, and possibly fecal-oral transmission [9]. One of the important complications that occurs after infection with these viruses is viremia, a lifelong complication that often occurs after infection with these viruses, and their relationship with human diseases is under investigation [10]. The described features are summarized in the figure below (Fig. 1).

**Table 1:** Contaminating AVs have been found in many tissues and body fluids of the human population and their effects have been studied

	Tissues	Biological fluids
**Torque teno virus (TTV)**	Bile [11, 12] Liver [13] Cervical [2] Bone marrow [12, 13] Gut [14] Nasal [15] Fetal [16] Gastric [17] Placenta [16] Cervical lymph nodes [18]	Blood [19] Saliva [20] Urine [21] Fecal [22] Sweat [23] Semen [20] Plasma [24] Breast milk [5] Amniotic fluid [25]
**Torque teno mini virus (TTMV)**	Bile [12] Liver [26] Cervical [2, 27] Bone marrow [12] Brain [28, 29] Gingival [30] Cervical lymph nodes [18]	Blood [24] Saliva [24]
**Torque teno midi virus (TTMDV)**	Cervical [31] Nasopharyngeal [32]	Blood [33] Urine [32] Stool [32]

TTV was the first virus of this family to be identified. It was first discovered in blood samples from a Japanese patient suffering from fulminant hepatitis. Its high prevalence in human populations indicates that this virus cannot be the leading cause of a particular disease. Still it has the ability to act as an auxiliary factor in the initiation and development of the disease [34, 35]. Fewer studies have been conducted on TTMV and TTMDV, but it is known that the prevalence of these viruses in human blood is significantly lower than in TTV [36]. Various studies have highlighted the relationship between the viral load of these viruses and various types of cancer, immunodeficiency diseases, and viral infections [10, 34, 37, 38]. It has also been stated that these viruses can interact with a person’s immune system and be used as a marker for the host’s immune system status in people suffering from immunodeficiency diseases or undergoing organ transplantation [39]. Also, it has been found that TTV is able to encode microRNAs (miRNAs) that are involved in viral evasion of the host immune response and regulation of viral reactivation and pathogenicity [40].

In this review article, we study and clarify the relationship between human anelloviruses and diseases that affect human health, and attempt to categorize and explain these related diseases.

## Overview of anelloviruses characteristics

Viruses of the Anelloviridae family have negative-sense single-stranded circular DNA genomes. Members of this viral family lack a double-layered lipid called the envelope around the viral capsid, and their virion shape is spherical with a diameter of about 30 to 50 nm. Also, the capsid of AVs consists of 12 pentameric capsomers with T = 1 icosahedral symmetry [21, 39, 41, 42]. Anelloviruses have an extensive prevalence worldwide, and reports and studies have shown their prevalence in humans and a wide range of animals, including mammals such as cattle, pets, primates, wild boar, sea lions, etc. [43]. Several genera and species of AVs that can infect a person are called anellome [44]. Although AVs are abundant in the human population, no appropriate cell culture or animal models are available for them, and this has limited our knowledge of these viruses [45]. However, it is possible to identify AVs through the PCR method [46]. Their genomic structure is similar to each other, but they differ in terms of genome length. The TTV genome is 3.5 to 3.9 Kb in length, whereas the TTMDV genome is up to 2.3 Kb, and the TTMV genome is 2.7 to 2.9 Kb [1, 47]. TTV has diverse tissue tropism, and the virus genome has the ability to replicate simultaneously in several different tissues and organs, such as the liver, spleen, pancreas, bone marrow, muscle, lung, kidney, peripheral blood mononuclear cells (PBMC), etc [21, 48]. However, T lymphocytes have been introduced as the principal place to multiply these viruses [49]. Their replication occurs in the cell nucleus and their replication mechanism is circular. They probably use cellular polymerases for their genome replication process [8, 47, 50].

**Fig. 2:**
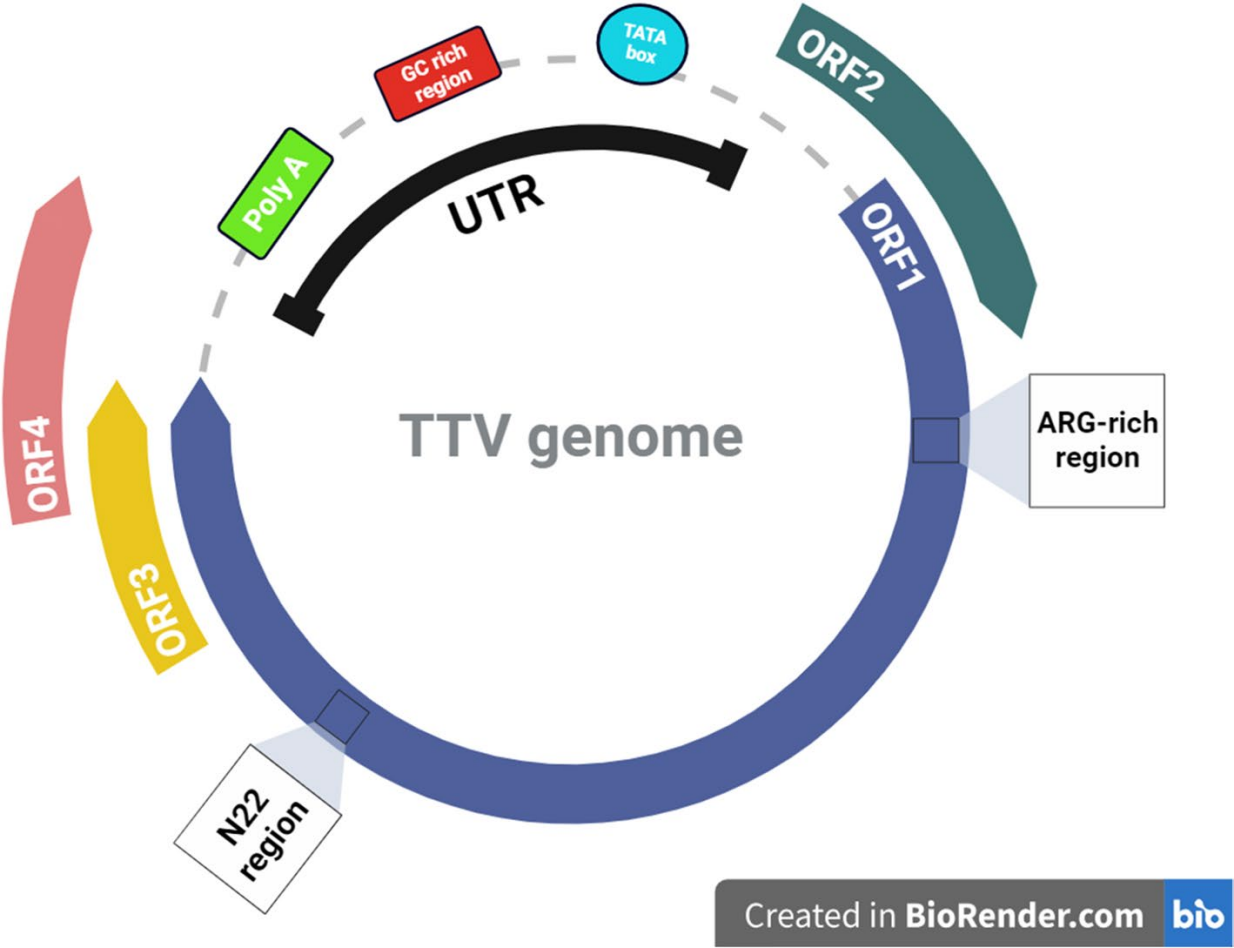
The genome of Torque teno virus. It consists of 3 to 5 ORFs and a non-coding UTR region that performs regulatory roles. Adapted from “Genome of Torque teno virus”, by BioRender.com (2023). Retrieved from https://app.biorender.com/biorender-templates

The viral genome is divided into two distinct main regions. These two areas are; The coding region of the genome and the non-coding region of the genome [8]. The untranslated region (UTR) of the virus genome creates hairpins using the sequences it contains to facilitate replication of the genome by the rolling circle method [51]. This region is divided into two parts, UTRA and UTRB, where UTRA is located in the 5′ region, and UTRB is located in the 3′ region, both of which are rich in GC bases [34]. GC-rich sequences in this non-coding region facilitate viral replication [16]. The genome of AVs contains 3 to 5 main open reading frames (ORFs) that are able to encode six or seven different proteins. These proteins weight 12 and 80 kDa and the largest one is ORF1 (Fig. 2). These viral ORFs overlap and interfere with each other to some extent. ORF2 and ORF3 have the ability to produce several viral proteins through alternative splicing mechanisms [50, 52–54]. ORF1 encodes the viral capsid protein, which is the largest protein of these viruses [55]. According to the location of an arginine-rich region in the N-terminal ORF1 of TTV, which is similar to the ARM motif that has the role of binding to the viral genome, it is speculated that the ORF1 protein plays a role in genome packaging and replication [51, 56]. Also, ORF1 is susceptible to more mutations due to the presence of hypervariable regions that help the virus escape from the host’s immune system [16]. Anelloviruses have been classified into three separate genera based on the difference in the ORF1 sequence [49]. Also, the ORF2 protein has the ability to suppress the canonical and non-canonical NF-κB pathways, which strengthens the possibility that the protein helps the virus to adapt to the host environment [57]. Inhibition of this pathway disrupts the translation of genes, and it causes a decrease in the production of inflammatory cytokines (IL6 and IL8) [58]. Also, the ORF2 region is divided into two smaller regions named ORF2a and ORF2b, which may be differ in different isolates [59]. During TTV (AV index virus) replication, three mRNAs are produced, which can eventually produce at least six proteins [60]. The virus probably interferes with the host cell life cycle using ORF3 [61]. ORF3 protein contains a region rich in serine amino acids, with groups of arginine and lysine amino acids in its C terminus. This protein contains several nuclear targeting sequences. This case suggests ORF3 is probably a transcriptional regulatory nuclear protein [62]. It is also homologous to DNA topoisomerase I, which strengthens the possibility that ORF3 also plays a prominent role in viral replication [63]. Other production proteins include; ORF2/2, ORF1/1, ORF2/3, and ORF1/2. The first two are produced by 1.2 kb mRNA, and the second two by 1 kb mRNA [51, 60]. Also, the virus can cause apoptosis in cancer cells through a protein encoded by itself, called TTV-derived apoptosis-inducing protein (TAIP) [61].

## The role of TTV-miRNA in human disease

miRNAs are small 22-nucleotide non-coding RNAs, the largest family of small non-coding RNAs that play the role of post-transcriptional regulation of gene expression and are produced in all higher eukaryotic cells [64–66]. In this way, they increase the degradation of coding mRNAs and regulate protein production [67]. Viruses have been able to develop various molecular mechanisms to target and inhibit the antiviral mechanisms of host cells. Viral miRNA encoding is one such mechanism used to regulate the expression of host and viral genes and is essential for the continuation of the virus life cycle [68]. miRNAs that are encoded by viruses are called viral miRNAs (v-miRNAs). To date, nearly 500 precursors or mature v-miRNAs have been identified and documented [69]. v-miRNAs are involved in pathogen-host interaction, immune evasion, tumorigenesis, and cell death during infection of host cells by viral agents [70]. In 2004, the first v-miRNAs were discovered in a herpesvirus called Epstein-Barr virus (EBV). As an example of the pathogenic role of viral miRNAs for viruses, it can be mentioned that EBV miRNAs can be dysregulated during blood malignancies [71, 72]. We should also mention the role of human cytomegalovirus miRNA (hcmv-miRNA) in the development of cardiovascular diseases or their oncogenesis by inhibiting tumor suppressors [73]. Hepatitis C (HCV) miRNAs can also be related to the pathogenesis of this infection and the resulting liver disease [74]. After nearly 20 years, the number of viruses capable of encoding and expressing miRNA has reached 20 [71].

During recent studies, it has become clear that TTV is also one of the viruses that have the ability to encode and produce its viral miRNAs. By interacting with viral proteins, these miRNAs regulate viral genes involved in processes such as pathogenesis, immune evasion, inflammation, and replication [16]. Among the miRNAs encoded by TTV, miRNA t1a, t3b, and tth8 have been found in the biological fluids of healthy people or those with certain diseases. Based on what previous studies show us, the aforementioned v-miRNAs can be identified and reported in both viremic and non-viremic individuals. ttv-miRNAs are involved in escaping from the host's immune system, viral reactivation, as well as pathogenicity, and finally maintaining chronic viral infection and helping the virus in these processes [65, 75, 76]. TTV uses the cellular miRNA-making machinery to encode and synthesize its viral miRNAs and use them to activate or inhibit specific intracellular signaling pathways [77]. Probably, some of these miRNAs contribute to the persistence of the virus in the host cells and escape from the immune system by disrupting cytokine signaling [78]. Based on what research has shown, ttv-miRNAs probably reduce the response to the cytokine IFN, and as a result, this activity can increase cell proliferation in the presence of IFN. It has been suggested that ttv-miRNA can target the transcript (mRNA) of the N-myc interacting gene (NMI). Targeting the transcript of this gene inhibits the interferon signaling pathway, resulting in no active immune response. Therefore, the virus can inhibit the interferon response and cause the immune system to escape. This is the result of the manipulation of the immune system by the virus [21, 79–81]. In reports, ttv-tth8-miR-T1 is mentioned as an IFN inhibitor that targets the interaction of N-myc and STAT to do this [82, 83]. In addition, miRNA-t3b can possibly continue the life cycle of the virus in the cell and escape from the immune system by targeting IL6 signaling and disrupting it. Based on observations, this process will probably be effective in the mortality of older people [78]. Also, studies have stated that TTMV miRNAs may be able to maintain cancer cells and facilitate their development by inhibiting interferon and thus modulating the immune responses of T cells [28]. In recent research, ttv-miRNA has been detected in small extracellular vesicles (EVs) of plasma from subjects with hepatitis B virus (HBV) and HCV infections, human immunodeficiency virus (HIV), solid organ recipients, and healthy people [76, 84]. It has also been proven that ttv-miRNA can modulate the host's immune system in people with multiple sclerosis and lupus [75]. However, despite all this, it is impossible to make a definite statement about the direct role of these viruses and their miRNAs in human diseases or even causing the disease by themselves, and the need for more research is felt.

## Human Cancer

According to recent WHO reports, viral infections are responsible for more than 10% of human cancers [85]. Today, seven human viruses that are directly involved in carcinogenesis have been identified. They include EBV, HBV, HCV, human papillomavirus (HPV), Kaposi sarcoma-associated herpesvirus, human T-cell lymphotropic virus 1, and Merkel cell polyomavirus [86]. Viruses apply their oncogenic strategies in both direct and indirect ways. In direct mode, the viral oncogenes are placed in the vicinity of cellular genes, or they apply changes to cellular proto-oncogenes and create mutations. In an indirect form, chronic and long-term infection and inflammation can lead to the emergence of cancer cells [87, 88].

Numerous studies have shown that changes in the composition of the human virome (called the viral part of the microbiome) are associated with malignant and oncogenic diseases [89]. Scientific findings show that high viral loads can be involved in the initiation or progression of human cancers [10, 90]. Unlike viruses such as adenoviruses, which have the ability to induce the S phase of the host cell cycle to aid in the replication of their genomes, anelloviruses do not, but can replicate rapidly in their host cells [91]. Also, based on experimental findings, the presence of AVs can highlight the process of inducing cell proliferation or oncogenic space created by oncogenic viruses. However, it is impossible to comment with certainty about its possible role in these processes [92, 93]. Viral miRNAs can inhibit the interferon signaling pathway in the host cell, and this process is probably associated with changes in cell proliferation [79]. It has been proposed that continuous infection with these viruses can increase certain chromosomal translocations in the genome of host cells, which can be an important factor in the development of lymphoma and leukemia [21, 94]. TTV infections typically do not show obvious clinical manifestations but can be associated with many organ dysfunctions [95]. The high circulation of this virus among the human population indicates that TTV is not the main cause of a specific disease. Still, TTV can help develop a disease as an auxiliary factor or cofactor [34]. It has been shown that TTV viral replication and, thus, viral load is significantly increased in patients with pathological conditions such as untreated solid cancer [96]. It has also been found that increased viral titers of TTV and HBV are associated with liver cancer in patients with this disease [97]. TTV infection in patients with HCV is probably associated with the occurrence of Hepatocellular carcinoma (HCC) in these patients, and this virus is also known to be a carcinogenic agent in certain cases of head and neck cancer (HNCC) [92, 98, 99]. In other studies, it was observed that the viral load of TTV in people with colorectal cancer shows a significant increase compared to healthy people, but it is not possible to comment on its relationship with this cancer [100, 101]. The coinfection of TTV with HPV can probably be a triggering factor in the development of cervical cancer. Also, TTV genotype 1 in coinfection with HPV is associated with poor prognosis of laryngeal cancer [35, 102]. In some cases, TTV was also detected in human lip and kidney tumors [103]. In studies, TTV load in women with breast cancer showed a significant increase compared to healthy women [104]. The DNA of TTV has also been identified in the neoplastic tissue of lung cancer and brain tumors, but it is not possible to talk about their relationship with certainty. For example, in a sample of glioblastoma tumors, two sequences of the genome of this virus were identified, and changes in the load of TTV in patients with lung cancer without idiopathic pulmonary fibrosis (IPF) have been considered possibly related to tumor growth. The amount of TTV in the PBMC of people who had cancer with various malignancies was reported to be higher than in the blood of healthy people [21, 105–107]. The presence of TTV DNA in lymphocyte cells, as well as B-cell lymphoma and Hodgkin’s lymphoma, has also been confirmed, which could indicate a significant association [108]. Fewer studies have been conducted on other anelloviruses that infect humans, namely TTMV and TTMDV, and unfortunately, few reports of their prevalence are available [21, 109]. In addition to TTV, TTMV has also been detected in the serum of Hodgkin’s lymphoma patients, and its prevalence is higher in these patients than in healthy individuals [110, 111]. In the studies conducted on brain tumors, two new strains of TTMV were isolated from brain tumor tissue metastasized from skin melanoma. But whether these viruses originate from the blood or the primary skin tumor is unknown [28]. Recent studies have also mentioned that TTMV can be involved in the pathogenesis of acute promyelocytic leukemia (APL) [112]. Like TTV, TTMV has been identified and reported in cervical tumors [90].

## Immune system diseases

The role of viruses, including human anelloviruses, in modulating their host’s immune system and contributing to the inflammatory process has been established [113]. It has been shown that TTVs interact with a class of immune molecules called pathogen-associated molecular pattern (PAMP) that recognized by pattern-recognition receptors (PRRs), which ultimately activate immune and inflammatory responses [113, 114]. Human anelloviruses have established a suitable interaction with the host’s immune system, and studies have shown that the speed of replication of these viruses can be an appropriate measure to monitor the overall function of the host’s immune system. More clearly, it can be said that TTV indicates the state of the host’s immune system. However, considering that there is little information about the other two viruses, TTMV and TTMDV, in immunosuppressed patients, stronger conclusions can be made by measuring the viral load of these two viruses in the immunosuppressed state and examining them [1, 115–117]. Disorders of immunosuppression can provide opportunities for increased TTV replication, and for this reason, the amount of this virus is used to monitor the immune status of transplant patients [96, 118]. These results show that, in general, a high TTV load indicates that the probability of graft rejection is low, but a low load can be associated with acute graft rejection [119]. This feature can be used for risk classification in human lung and kidney transplantation [120]. It has been said that the rate of TTV proliferation is directly related to the age of the host; that is, with the increase of the age of the host, the multiplication of TTV in the host cells is likely to increase. The increase in the amount of virus load in the host is related to the decrease in the quality of the host’s immune system due to aging. It is possible that this increase in viremia can lead to immune system disorders and increased mortality in the elderly population [19, 121]. Also, TTV has beneficial effects on the immune system of infants, which will ultimately cause the growth and maturation of their immune system [7, 122]. A viral protein produced from ORF1 may direct and control the escape of these viruses from the host’s immune system [8, 123]. TTV load is also significantly increased in patients with autoimmune inflammatory diseases [124]. People infected with TTV can probably get autoimmune diseases or have their severity affected due to the effect of this virus on the host’s immune system and its modulation [125, 126]. Also, the increase in TTV DNA load in people with systemic lupus erythematosus, which is an autoimmune disease, is significant compared to healthy people. It has been shown that the TTV epitopes identified in these patients are probably involved in the pathogenesis of this virus in Systemic lupus erythematosus (SLE) patients [127, 128]. In one observation, the burden of TTV in patients with rheumatoid arthritis (RA), which is considered an autoimmune disease, was much higher than in healthy subjects, and it was suggested that TTV could be related to this disease. However, it is unclear whether this relationship is direct or indirect [21, 129]. Also, in patients with idiopathic inflammatory myopathy, infection with this virus may affect the severity of the disease in affected individuals and increase it [130]. TTV can even be involved in the development or maintenance of the autoimmune disease of Multiple sclerosis (MS) through the expansion of T cells derived from MS patients, and the possible role of different types of TTMV in MS has also been considered [131, 132]. In the case of non-A-E hepatitis, based on the investigations, it is possible that TTV acted as a cofactor for the immune attack on the liver, which resulted in severe liver damage as a result of this attack [133]. The course of Kikuchi–Fujimoto disease (KFD) suggests that it is probably caused by an immune response to a mostly viral infectious agent such as TTV/TTMV [134].

Inflammatory bowel disease (IBD) refers to conditions that lead to chronic or recurrent inflammation of the digestive tract. It includes several different disorders, including Crohn’s disease (CD) and ulcerative colitis (UC) [135–137]. Until today, the factors involved in causing these disorders remain unknown [137]. It is said that the human gut microbiome, which consists of bacteria, viruses, and fungi, is involved in the pathogenesis of this disease. The gut microbiome forms a complex ecosystem. Various activities are carried out by these microorganisms in the host, including Metabolic function, immunity, etc [138, 139]. In this disease, the immune system’s irregular response to the intestinal mucosa leads to chronic inflammation, which ultimately leads to tissue damage [140]. Recent studies emphasize the role of microorganisms in the pathogenesis and progression of IBD [141]. Significant changes in the population composition of the intestinal virome can play an effective role in the initiation and development of IBD, which is associated with chronic and systemic immune disorders [142]. An increase in anellovirus sequences has been observed in patients with IBD, strengthening the hypothesis that they may indirectly contribute to the progression of the disease [143]. In patients with CD, it was observed that TTV is related to inflammation in this disease, and relationships were found with complications and treatment approaches [144]. AVs are elevated in patients with IBD before initiation of therapeutic approaches. In studies conducted on patients with CD, several species of this family have been found in the feces of people with CD [145].

## Viral infection

Due to the common routes of transmission with HIV and HBV, coinfection of TTV with these viruses is common [46]. For the first time, TTV was detected in the blood of a Japanese patient who was infected with HBV [146]. TTV is most commonly seen in patients suffering from viral hepatitis. TTV appears to be associated with viral diseases that involve the liver, including HBV and HCV. Based on research, TTV can participate in coinfection with HBV or HCV [42, 147, 148]. In the coinfection of TTV with HCV, TTV can interfere with the process of liver disease caused by HCV and increase its severity in the sick host [149]. In recent studies, it has been clarified that the occurrence of HCC in people with chronic HCV infection who are simultaneously infected with anelloviruses can be related to anellome [44]. Higher levels of human anelloviruses have been observed in HIV-1 infected patients, including increased TTV load in these patients [19, 150, 151]. The increase in TTV load in patients with HIV indicates the status of the immune system of these patients [152]. In patients with HIV, the variety and frequency of AVs increases due to the occurrence of immunosuppressed conditions [153]. TTV is related to the status of T-CD4+ immune cells in HIV patients [2]. In the infection caused by HIV, with the decrease of T-CD4+ lymphocyte cells, the opportunity to increase TTV load is provided, and they increase significantly in the person with human immunodeficiency syndrome [154]. The amount of viral load of TTV in the plasma of people with AIDS shows a noticeable decrease after starting the treatment of these patients [155]. TTV-specific miRNAs were observed in the plasma and cerebrospinal fluid of patients infected with HIV, HCV, or HBV [65]. Also, high TTMV DNA load has been observed in tissues such as bone marrow and spleen of people who are HIV positive or have progressed to AIDS [12]. TTMDV has also been reported in people infected with HBV, HCV, and HIV pathogens, but it is not possible to speak with certainty about its pathogenicity in these patients. The frequency of TTMDV in patients with HIV, as well as patients with HCV and HIV coinfection, has a significant difference compared to healthy people [33]. Also, coinfection with AV and HPV has been shown in various studies [156]. TTV is more common in patients with high-risk HPV types than in patients with low-risk HPV types [2]. In this context, studies have shown the presence of TTV in patients with HIV/HPV coinfection [157]. In the conducted research, it has been clarified that the amount of TTV load has increased in patients who are simultaneously infected with EBV, and this may be effective in the subsequent effects of EBV in patients [158]. Research results have shown that high TTV DNA load is associated with persistent HCMV infection, especially in younger individuals [159]. Members of the anelloviride family are among the viruses whose high incidence has been proven in the respiratory system of people with acute respiratory viral infections, and it has been stated that their diversity is more significant in these patients than in healthy people [160]. Examining TTV DNA load in the plasma of people with respiratory disease COVID-19 who were infected by the SARS-CoV2 virus showed that this monitoring can be useful for predicting severe infections and mortality of these patients [161]. In studies of patients with COVID-19, it was found that a ttV-miRNA called tth8 could possibly affect the severity of the disease [65]. It has been found that TTV is implicated in the increased mortality associated with COVID-19 in cancer patients [162].

## The role of anelloviruses in organ transplantation

To prevent rejection of the transplanted organ in patients undergoing solid organ transplantation (SOT), immunosuppressant treatment is prescribed for them. However, treatment with these drugs increases the possibility of infections in patients. Therefore, finding a reliable safety status indicator for these receivers is very important [58]. The host’s immune system chronically controls AVs infecting humans, and their viral load increases in the blood if the immune system is suppressed. In recent years, it has been proven that reducing the viral load of these viruses is related to the rejection of solid organ transplants. As a result, the number of DNA copies is considered a suitable indicator for measuring the immune status of patients [81, 163–165]. It has been shown that they can be used to predict the possible risk of organ transplant rejection and the appearance of bacterial infections in these patients [166]. In the meantime, TTV has received more attention than the other two viruses. Its unique features, such as high prevalence among the population, global prevalence, stable viral loads with low intra-individual variability, resistance to antiviral drugs, and the use of quantitative PCR assays for its detection, highlight its role as an indicator of immune status [119].

TTV viral load is associated with short-term and long-term lung transplant outcomes and is known as a biomarker of immune system status in lung transplant recipients [167]. Probably, low levels of TTV in lung transplant recipients cause the occurrence of acute transplant rejection, which is considered a short-term outcome. Also, low levels of TTMV in these patients are associated with long-term consequences that can ultimately lead to death within two years [37]. Also, in the studies conducted on people with acute exacerbation of idiopathic pulmonary fibrosis (IPF), TTV was isolated from about 1/4 of the bronchoalveolar lavage (BAL) samples of these people [155, 168]. Studies have shown that TTV viral load are associated with acute rejection in heart and lung transplant recipients. Its viral load begins to increase after heart transplantation and reaches its highest level within from 90 to 120 days. After transplantation, rejection was observed in patients with lower viral load than in patients who accepted the transplant. These findings can help to recognize TTV as a biomarker of immune status after heart transplantation [169, 170]. Also, in other studies on lung transplant recipients, the amount of TTV viral load only at the first peak after transplantation can be a suitable indicator for the immune status of these patients [171]. Studies conducted on children undergoing kidney transplants have shown that no relationship can be found between the predominance of the titer of a particular genus of this family and the possibility of kidney transplant rejection. However, the low titer of TTV and other AVs after kidney transplantation increases the risk of organ rejection [172]. As a result, measuring its viral load predicts organ transplant rejection in these patients [173]. ORF2 of TTV produces a protein called TTV-Ag, which can reduce the level of interleukin-6 in the blood of kidney transplant patients by interfering with the NF-kB signaling pathway, thereby regulating the adaptive and innate immune system. This strategy probably reduces the possibility of transplant rejection by the individual’s immune system [174]. Several studies have proven that after liver transplantation (LT), TTV viral load increases due to immunosuppression. This increase continues until three months after transplantation and then gradually decreases. This indicates a reduction in immunosuppressive drugs. All this shows that the viral load of this virus has a direct relationship with the intensity of suppression [147, 175, 176]. Studies have shown that in pediatric patients, TTV proliferation after liver transplantation is closely related to immune system status and hepatitis E (HEV) coinfection [177]. Monitoring TTV DNA load in the plasma of patients undergoing allogeneic hematopoietic stem cell transplantation (allo-HSCT) has also been described as helpful in evaluating the immune system status of these patients [117]. This type of transplant is a cell therapy to improve malignant and non-malignant blood disorders [178]. Post-transplant viral load kinetics in these individuals can be highlighted as a marker for immunologic reconstitution [119].

## Conclusion

Human anelloviruses, namely TTV, TTMV, and TTMDV, are newly discovered human viruses, various aspects of their life cycle are still unclear, and research on this virus is progressing slowly. In this review, we clarified that these viruses, especially TTV, are of special importance. Thier miRNAs have also been identified in coinfection with viruses such as SARS-COV-2. This text, investigates the identification and emergence of AVs in human cancers. Also, they have been seen in association with infections of important viruses such as HBV and HIV, and this can make the function of these viruses questionable. Therefore, the possibility of their interaction with other viruses and the result of this interaction may add to the complications of the infection. We investigated their presence in human immunodeficiency diseases and clarified this part of virus function. We also emphasized the importance of these viruses in organ transplantation and transplant rejection. However, they still have undiscovered dimensions that need further investigation.

## Data Availability

Not applicable.
